# Multi-band ultrathin reflective metasurface for linear and circular polarization conversion in Ku, K, and Ka bands

**DOI:** 10.1038/s44172-024-00266-5

**Published:** 2024-09-09

**Authors:** Humayun Zubair Khan, Abdul Jabbar, Jalil ur Rehman Kazim, Masood Ur Rehman, Muhammad Ali Imran, Qammer H. Abbasi

**Affiliations:** 1 University of Glasgow, Glasgow, 12G8QQ UK; 2 National University of Sciences and Technology, Islamabad, 44000 Pakistan

**Keywords:** Electrical and electronic engineering, Physics

## Abstract

Linear polarization (LP) and circular polarization (CP) holds paramount importance in Ku, K, and Ka bands for satellite based communication, and remote sensing applications. Satellite based remote sensing applications face challenges like atmospheric attenuation, noise & interference, and signal degradation. Moreover, satellite based communication application demands CP in two distinct, non-adjacent frequency bands with orthogonal polarizations at greater oblique angles, considering the unpredictable incidence angles of electromagnetic (EM) waves. Addressing these challenges, an innovative metasurface polarization converter is proposed to operate efficiently across the Ku-band (13.5–18.0 GHz), K-band (18.0–26.5 GHz), and Ka-band (26.5–38.5 GHz) frequency ranges. The converter achieves left-handed circular polarization (LHCP) in the Ku- and Ka-bands within the frequency ranges of 14.57–15.65 GHz and 27.47–33.85 GHz for **y**-polarized incident EM waves. Additionally, it provides right-handed circular polarization (RHCP) in the K- and Ka-bands at 17.27–23.92 GHz and 35.87–38.32 GHz for **y**-polarized incident EM waves. The LP conversion ratio exceeds 95% in the frequency bands of 15.97–16.85 GHz, 24.70–26.65 GHz, and 34.37–35.45 GHz for **y**-polarized, LHCP, and RHCP incident EM waves, respectively. The metasurface exhibits robust performance up to incidence angles of 45 degrees under oblique conditions. Experimental validation using traditional board-circuit manufacturing demonstrates close agreement between measured co- and cross-polarized reflection coefficients and simulations in the 13.5–18 GHz, and 24–38.5 GHz frequency range. Thin metasurface with a thickness of only 0.64 = 0.013*λ*_*o*_ mm, the proposed design outperforms existing studies in the literature, establishing its competitive edge in terms of structure and performance.

## Abstract (web summary)

Humayun Zubair Khan and colleagues design a thin and efficient metasurface for polarisation conversion. Their solution can operate to up to 45-degree incidence angles and is suitable for satellite communications which use separated frequency bands with orthogonal circularly polarised waves.

## Introduction

Metasurfaces have become pivotal in the evolution of satellite communication (Satcom), and satellite technologies, particularly in the Ku, K, and Ka frequency bands^1^. Manipulation and control of the polarization state have many applications in wireless communication systems, such as satellite communication, and remote sensing^2^. The significance of metasurfaces lies in their unparalleled ability to manipulate electromagnetic (EM) waves with precision, addressing the unique challenges posed by these high-frequency bands. In the realm of communication, these bands are vital for satellite communication (Satcom) and high-speed data transmission^3^. Additionally, polarization conversion in satellite remote sensing enhances data accuracy by reducing atmospheric interference, improving image resolution, and enabling better discrimination of surface features, crucial for environmental monitoring, agriculture, and disaster management^4^. Hence, it becomes evident that their role extends beyond technological innovation - they serve as key enablers, unlocking new frontiers in wireless communication, remote sensing, and satellite technology.

Linear-to-linear polarization (LLP) conversion is crucial for optimizing Satcom and satellite remote sensing across the Ku, K, and Ka frequency bands. Linear polarization (LP) finds extensive use in Satcom and satellite remote sensing, bolstering communication link reliability and data integrity. This capability supports a broad spectrum of applications, from high-speed data transfer to Satcom^5^. In vegetation analysis, LP aids in distinguishing between different vegetation types based on their distinct polarization signatures. By measuring vegetation’s polarization response, remote sensing satellites can evaluate vegetation density, health, and distribution, facilitating ecological studies and agriculture monitoring^6^. Moreover, LP proves effective in identifying smoke particles in the atmosphere by analyzing their depolarizing effects, enabling early wildfire detection and air quality monitoring^7^.

Linear-to-circular polarization (LCP) conversion stands as vital as LLP in advancing Satcom and satellite remote sensing technologies across the Ku, K, and Ka frequency bands^8–10^. Circular polarization (CP) enhances signal robustness, tackling issues like signal fading and interference^11^. In Satcom applications, it is crucial for antennas not only to exhibit CP but also to operate in two distinct, non-adjacent frequency bands with orthogonal polarizations^12^. The Canadian RADARSAT Constellation Mission (RCM) utilizes a circular polarized synthetic aperture radar (SAR) architecture, transmitting a right-hand circular polarized (RHCP) signal and receiving both vertical and horizontal backscattered components. Its applications span from sea ice mapping and ocean target detection to wind speed estimation and marine pollution monitoring^13^.

Having established a comprehensive understanding of LP, CP, and their wide-ranging applications in Satcom and satellite remote sensing, the literature review now explores the emerging field of reflective metasurfaces for LP and CP conversion across the Ku, K, and Ka frequency bands. This innovative avenue promises ground-breaking advancements in antenna technology, paving the way for enhanced signal manipulation and improved performance in communication and remote sensing systems.

For example, in^14^, researchers designed a metasurface featuring a straightforward square with two curved cuts, achieving narrow-band LP with commendable Polarization Conversion Ratio (PCR) and Angular Stability (AS), along with narrow-band CP (LHCP, RHCP) with decent Fractional Bandwidth (FB) for Ku band applications. Similarly,^15^ accomplished broad-band LP with favorable PCR and AS, and narrow-band CP (LHCP only) with reasonable FB in reflection mode for Ku band applications.

In^16^, authors presented a versatile metasurface achieving wide-band LP with commendable PCR and AS, and narrow-band CP (LHCP only) with low FB for applications in X and Ku bands. Researchers in^17^ proposed a metasurface structure realizing narrow-band LP with good PCR and AS, and narrow-band CP (LHCP, RHCP) with satisfactory FB for applications in C, X, and Ku bands.

In the domain of C, X, and Ku bands,^18^ introduced a multi-band meandered square ring metasurface accomplishing narrow-band LP with notable PCR and low AS, and narrow-band CP (LHCP, RHCP) with fair FB, making it suitable for satellite, radar, and 5G communication applications.^19^ reported a symmetric anisotropic metasurface achieving narrow-band LP with commendable PCR and low AS, and narrow-band CP (LHCP, RHCP) with fair FB in reflection mode for C, X, and Ku band applications. Within the Ku and K bands,^20^ proposed an innovative metasurface featuring a multi-band diagonally split circular ring metasurface that achieves broad-band CP (LHCP, RHCP) with favorable FB and AS that is well-suited for applications in Satcom, radar systems, and 5G communication networks.

Likewise, in the C and X bands,^21^ designed a metasurface achieving wide-band LP with good PCR and commendable AS along with wide-band CP (LHCP, RHCP) with good FB and AS for applications in X, Ku, and K bands. In ref. ^22^, authors demonstrated metasurfaces achieving wide-band CP (LHCP, RHCP) with commendable AS and FB for Ku, K, and Ka band applications.^23^ proposes a dual-band dual LP-to-CP converter for Ka-band satellite communications, operating in transmission mode. It consists of a single 1.05 mm thick panel, generating LHCP at lower band and RHCP at higher band with AS up to 45 degree in K, and Ka bands.^24^ introduces a dual-band reflective LP to CP converter that achieves RHCP in the lower band and LHCP in the higher band with AS up to 30 degree. Experimental results validate simulated performance. A summary of past work is provided in Table 1.

**Table 1:** Comparison table with state of the art literature

Ref	Band	LP	PCR	AS	LHCP	FB in LHCP	RHCP	FB in RHCP	Length (mm)	Thick (mm)
[14]	Ku	15.5-16.5	≥85%	≤60^*o*^	17.4–18.35	5.31%	12.88–13.57	5.21%	6 = 0.280*λ*_*o*_	2 = 0.093*λ*_*o*_
[15]	Ku	12.05-17.9	≥80%	≤45^*o*^	11.3–11.5	1.7%	-	-	6.6 = 0.308*λ*_*o*_	1.2 = 0.056*λ*_*o*_
[16]	X, Ku	8.32-17.55	≥93%	≤45^*o*^	7.42–7.68	3.44%	-	-	7.8 = 0.32*λ*_*o*_	2.57 = 0.10*λ*_*o*_
[17]	C, X, Ku	6.46-6.78 10.52-11.85 16.49-17.37	≥90%	≤45^*o*^	7.28–9.40	25.4%	13.38–15.19	12.6%	8 = 0.30*λ*_*o*_	1.60 = 0.061*λ*_*o*_
[18]	C, X, Ku	4.3, 7.2,12.3 15.15	≥90%	≤15^*o*^	4.75–5.95 14.35-14.6	22.4%1.7%	8.35–8.8	5.2%	11 = 0.333*λ*_*o*_	3.2 = 0.098*λ*_*o*_
[19]	C, X, Ku	7.2-10.51 15.81-17.48	≥90%	≤30^*o*^	12.54–13.76	9.2%	6.78–6.89 18.39-18.82	1.6% 2.3%	7 = 0.291*λ*_*o*_	2.4 = 0.1*λ*_*o*_
[20]	Ku, K	-	-	≤45^*o*^ ≤ 30^*o*^	15.25–18.9	21.37%	29.7–36.7	21.08%	5.20 = 0.45*λ*_*o*_	0.3 = 0.26*λ*_*o*_
[21]	X, Ku, K	12.35-17.8	≥80%	≤50^*o*^	10.85–11.05 21.85-27.40	1.82% 22.53%	12.35–17.8	36.15%	5.94 = 0.35*λ*_*o*_	1.6 = 0.096*λ*_*o*_
[22]	Ku, K, Ka	-	-	≤45^*o*^	15–21.2	34.3%	27–30.3	11.5%	2.8 = 0.23*λ*_*o*_	1.032 = 0.169*λ*_*o*_
[23]	K, Ka	-	-	≤45^*o*^	19.7–20.2	2.50%	27–30.3	1.68%	5.3 = 0.44*λ*_*o*_	1.05 = 0.08*λ*_*o*_
[24]	C,X,Ku	-	-	≤30^*o*^	10.3–14.73	35.39%	8.34–8.71	4.34%	11 = 0.45*λ*_*o*_	1.6 = 0.06*λ*_*o*_
**This work**	**Ku, K, Ka**	**15.97–16.85 24.70–26.65 34.37-35.45**	≥**95**%	≤**45**°	**14.57–15.65 27.47–33.85**	**7.14% 20.8% (27.94%)**	**17.27–23.92 35.87–38.32**	**32.3% 6.60% (38.90%)**	**2** = **0.17λ**_**o**_	**0.64** **=** **0.05***λ*_**o**_

The length (mm) is the length of metasurface in relation to *λ*_∘_. The thick (mm) is the thickness of metasurface in relation to *λ*_∘_ = *c*/*f*_∘_, and *f*_∘_ = 26 GHz.

In Satcom applications, it is crucial for antennas not only to exhibit CP but also to operate in two distinct, non-adjacent frequency bands with orthogonal polarizations^25^. This ensures enhanced isolation between transmitted and received signals, particularly in environments with low power flux densities susceptible to real-time interference^12^. Furthermore, it is essential for polarizer metasurfaces to perform effectively at greater oblique angles, considering the unpredictable incidence angles of EM waves. This capability becomes increasingly important at higher frequency ranges, where destructive interference may occur due to the extended path traveled by the electromagnetic wave within a dielectric spacer^26^. Addressing these challenges and drawing inspiration from prior research^14–24^, this study proposes a reflective metasurface that achieves following:

1. The proposed metasurface demonstrates LLP conversion with bandwidths of 0.88 GHz (Ku band), 1.95 GHz (K band), and 1.08 GHz (Ka band) with resonance at 16.37 GHz, 25.75 GHz, and 34.92 GHz, respectively. The design achieves high efficiency, with a PCR exceeding 95% and AS up to 45°.
2. The proposed metasurface exhibits the LCP in four distinct bands with bandwidths of 1.08 GHz (Ku-band, LHCP), 6.65 GHz (Ku, and K-band, RHCP), 6.38 GHz (Ka-band, LHCP), and 2.45 GHz (Ka-band, RHCP). The minimum band gap between adjacent bands is 1.62 GHz. Moreover, the proposed metasurface ensures a stable axial ratio (AR) and ellipticity at oblique incidences up to 45°.
3. To validate the proposed design’s performance, we fabricated and compared it experimentally with simulation results and observed a satisfactory agreement between experimental and simulated polarization conversion results.
4. The proposed metasurface was also modeled using an equivalent circuit, showcasing a notable concurrence between simulated and circuit-derived reflection coefficients, as well as AR. The successful correlation between simulation and the equivalent circuit enhances the reliability of the metasurface’s performance predictions.
5. This metasurface design showcases its adaptability for dedicated applications in Satcom, and remote sensing across Ku, K, and Ka bands, particularly where achieving LCP (with orthogonality) and LLP conversion is of primary concern.

## Results and discussion

### Unit cell

**Fig. 1:**
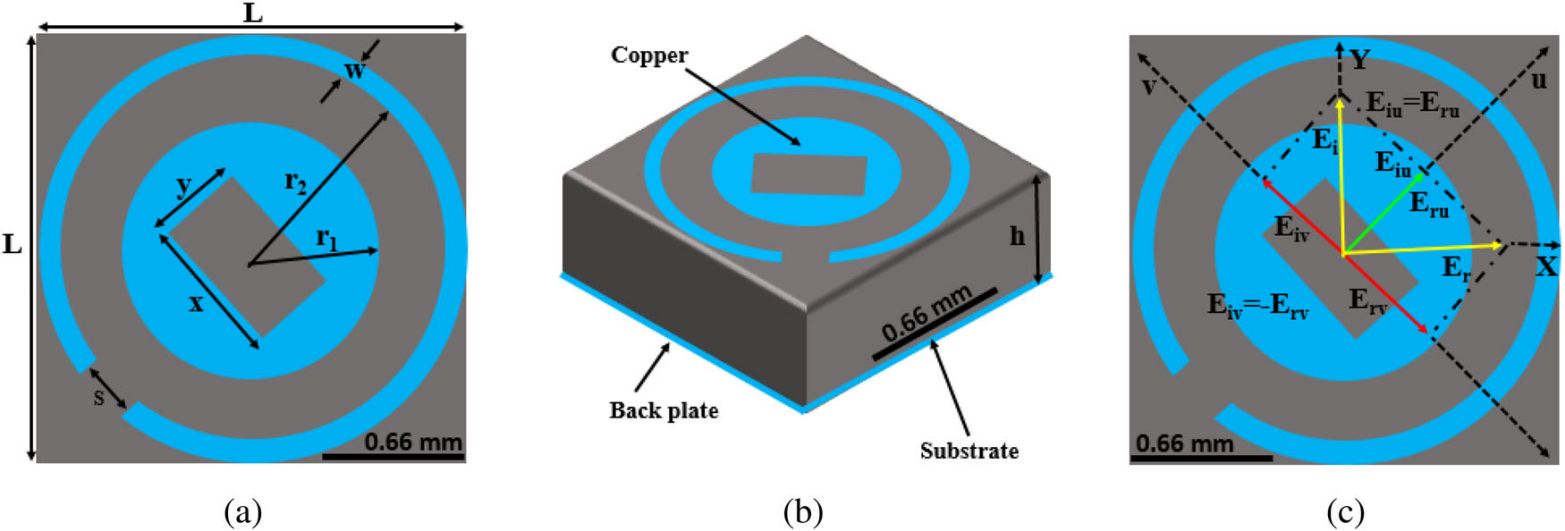
The unit cell design. **a** Front view of the unit cell (**b**) Perspective view of the unit cell (**c**) UV diagram of the unit cell.

The structural design of the proposed metasurface is depicted in Fig. 1, presenting the unit cell from three distinct perspectives: the front view (a), the perspective view (b), and the UV-diagram (c). The design involves three essential components: a top copper cell, an intermediate dielectric substrate, and a bottom copper plate. When an EM wave interacts with the structure, it produces x- and y-polarized transmitted and reflected EM waves. The ultimate reflected wave arises through multiple reflections between these transmitted waves and the lower metallic ground. Crucially, intentional control of the phase and magnitude of the reflected EM wave is achieved by regulating interactions within the dielectric and ground plane.

To ensure structural compactness, precise parameter selection, particularly in regard to the thickness of the dielectric spacer facilitating multiple reflections, is essential. The top and bottom layers are separated by a Rogers RO3003 substrate with a permittivity of *ϵ*_*r*_ = 3.00 ± 0.04 and a loss tangent of *δ* = 0.0010, having a thickness of *h* = 0.64 mm. The top and bottom layers consist of copper with an electrical conductivity of *σ* = 5.8 × 10^7^ S/m and a thickness of *t* = 0.035 mm. The optimized parameters of the unit cell are *r*_1_ = 0.55 mm, *x* = 0.35 mm, *y* = 0.12 mm, *r*_2_ = 0.83 mm, *s* = 0.10 mm, and *h* = 0.64 mm.

### Fabrication and experiment

**Fig. 2:**
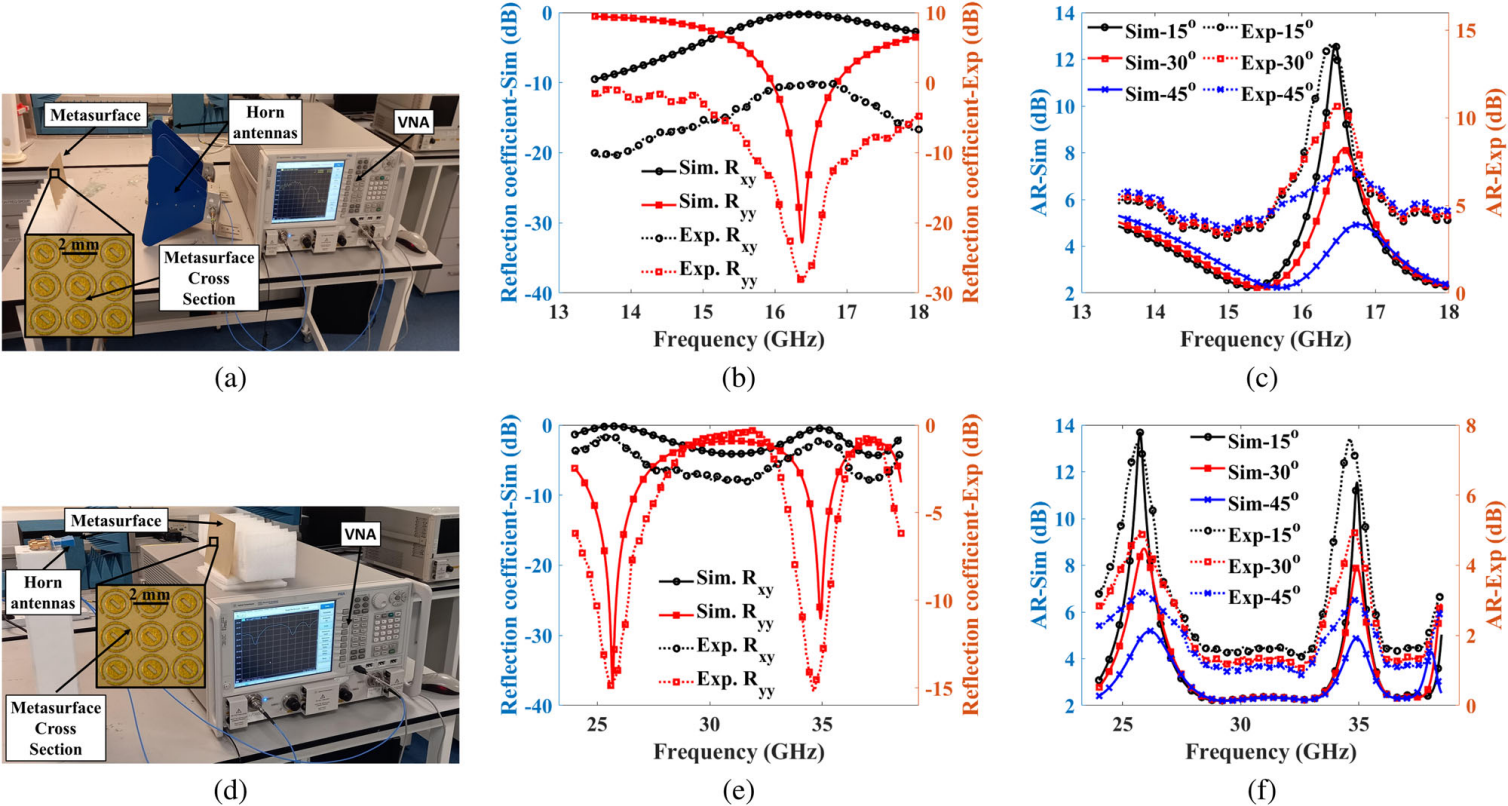
Free-space real-time measurements. **a** experimental setup for measurements between 13.5–18 GHz frequency range (**b**) simulated (Sim) and measured (Exp) co-reflection R_*yy*_ and cross-reflection R_*xy*_ coefficients between 13.5–18 GHz frequency range (**c**) Sim and Exp axial ratio between 13.5–18 GHz frequency range (**d**) experimental setup for measurements between 24–38.5 GHz frequency range (**e**) Sim and Exp co- and cross-reflection coefficients between 24-38.5 GHz frequency range (**f**) Sim and Exp axial ratio between 24-38.5 GHz frequency range.

Initially, we simulated the reflective metasurface’s co-reflection (R_*yy*_) and cross-reflection coefficients (R_*xy*_) using CST Microwave Studio^*Ⓡ*^. Subsequently, to validate the practical performance of our designed reflective metasurface polarization converter, we conducted experimental measurements on the fabricated prototype, as illustrated in Fig. 2. The fabricated prototype comprises 110 × 150 unit cells. Our experimental setup, depicted in Fig. 2a, d, utilized two wide-band horn antennas spanning frequencies of 2–18 GHz and 24–40 GHz, respectively, to illuminate the metasurface and capture reflected EM waves. The Agilent PNA network analyzer N5224A facilitated signal transmission and reception, employing TRL calibration for transmission line calibration.

For R_*yy*_ measurements, both antennas were aligned vertically, while for R_*xy*_ measurements, one antenna was positioned horizontally and the other vertically. To ensure accurate measurements, we maintained a proper far-field distance (d) between the metasurface and the Tx/Rx antennas. This distance was determined by the formula 2*D*^2^/*λ*_∘_, where D represents the metasurface’s dimensions and *λ*_∘_ is the free space wavelength at 23.5 GHz^27^. While increasing the distance results in higher path loss due to free space propagation, maintaining performance integrity, especially in polarization conversion across desired bands, is crucial. Our experiments revealed a negligible 2 dB decrease within a 10 cm range, indicating stable performance. Practical scenarios addressing path loss concerns may involve high-gain antennas or amplifiers, albeit beyond this study’s scope.

Figure 2b, e presents the co-reflection (R_*yy*_) and cross-reflection coefficients (R_*xy*_), both experimentally and in simulation, covering the frequency range from 13.8 to 18 GHz and 24 to 38.5 GHz. Throughout this spectrum, cross-reflection coefficients consistently remained above -1 dB, while co-reflection coefficients consistently stayed below −10 dB. This illustrates a close correspondence between simulated and experimental outcomes, acknowledging minor discrepancies attributed to fabrication imperfections and the inherent challenges associated with small-scale prototypes. Additionally, Fig. 2c, f displays simulated and experimental results for the AR in dB, spanning the frequency ranges of 13.8 to 18 GHz and 24 to 38.5 GHz, respectively. Notably, measurements for normal incidence were not taken due to limitations of the horn antennas. However, for incident angles of 15°, 30°, and 45°, a satisfactory agreement between experimental and simulated data is observed. A slight rightward shift in the central frequency is noticeable, possibly due to inaccuracies in the provided dielectric constant values.

A comparative analysis was conducted with existing works, including^14–24^. The focus was on the operating band, polarization type (LP, CP), PCR, AS, FB, and unit cell dimensions in terms of wavelength. The proposed metasurface converter stands out with a compact unit cell length of 2 = 0.17*λ*_*o*_ mm and a thickness of 0.64 = 0.05*λ*_*o*_ mm, where *λ*_*o*_ is central wavelength. This places the converter in a competitive position for applications requiring thin designs, such as antennas and radar cross-section^28–30^. Notably, the proposed converter maintains over 95% PCR even at higher incidence angles (up to 45°), outperforming the performance of previous studies^14–24^ In term of AS, the proposed converter performance aligns with^15–17,20,22,23^, and outperforms the work in^18,19,21,24^. In terms of LHCP, the proposed convertor outperforms the work in^14–21,23^. Similarly, in terms of RHCP, the proposed convertor outperforms the work in^14–20,22–24^. In summary, the proposed ultra-thin reflective metasurface excels in the Ku, K, and Ka bands, surpassing existing metasurfaces in the literature.

### Equivalent circuit model

**Fig. 3:**
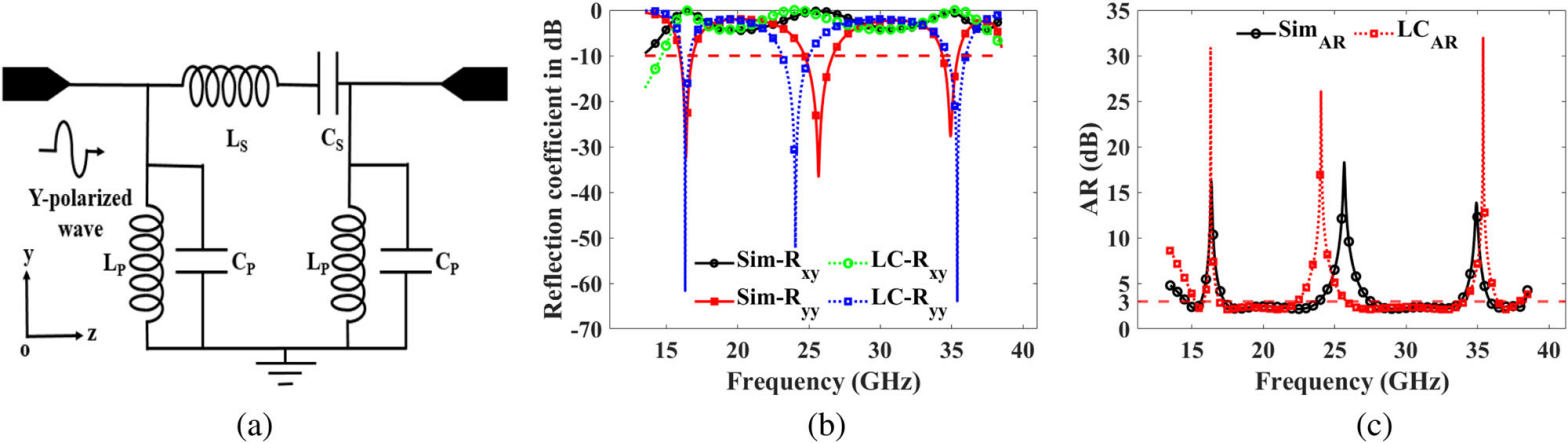
Equivalent circuit model. **a** the schematic diagram for equivalent inductor-capacitor (LC) circuit (**b**) co-reflection (R_*yy*_) and cross reflection (R_*xy*_) coefficients for simulations (Sim) versus LC circuit (**c**) axial ratio for Sim versus LC circuit.

The equivalent circuit model of metasurfaces represents the complex EM response of the metasurface structure using a simplified circuit-based approach. This approach allows for a more intuitive understanding of the metasurface behavior and facilitates analysis and design. The proposed metasurface was also modeled using an equivalent inductor (L) and capacitor (C) LC-circuit. The top surface of metasurface is modeled as a combination of one series LC circuit leg and two parallel LC circuit legs. The co-reflection (R_*yy*_) and cross-reflection coefficients (R_*xy*_) generated by this combination are close to the co- and cross-reflection coefficients generated by the proposed metasurface. The short circuit is taken to mimic the perfect ground for the reflection type metasurface. This combination represents Chebyshev band pass filter (BPF) circuit model as shown in Fig. 3a. In order to calculate the lumped elements at the desired operating frequency for the Chebyshev BPF prototype, the circuit must be scaled using below equations

1a $${{\mbox{L}}}_{{{{\rm{p}}}}}=\frac{{{\mbox{g}}}_{p}\Delta }{{{\mbox{w}}}_{\!\!\circ }{{\mbox{R}}}_{\!\circ }},{{\mbox{C}}}_{{{{\rm{p}}}}}=\frac{{{\mbox{g}}}_{p}}{\Delta {{\mbox{w}}}_{\!\!\circ }{{\mbox{R}}}_{\!\circ }}$$

1b $${{\mbox{L}}}_{{{{\rm{s}}}}}=\frac{{{\mbox{g}}}_{s}{{\mbox{R}}}_{\!\circ }}{{{\mbox{w}}}_{\!\!\circ }\Delta },{{\mbox{C}}}_{{{{\rm{s}}}}}=\frac{\Delta }{{{\mbox{g}}}_{s}{{\mbox{w}}}_{\!\!\circ }{{\mbox{R}}}_{\!\circ }},$$

where g_*p*_, g_*s*_ are the lumped element values, R_∘_ is the impedance value, w_∘_ is the center frequency, and Δ is the fractional bandwidth of the filter^31^. The values of the lumped components are tabulated in Table 2. The calculated lumped component values in (1a), and (1b) are used in the Keysight ADS^*Ⓡ*^ circuit simulator to plot the reflection coefficients (dB) and AR (dB). There is a good agreement between simulated and equivalent circuit reflection coefficients as shown in Fig. 3b. Similarly, a good agreement between simulated and equivalent circuit AR (dB) as shown in Fig. 3c. This design exhibits a promising alignment, highlighting the accuracy of the simulated results from the designed metasurface compared to the theoretically derived equivalent circuit model. The successful correlation between simulation and the equivalent circuit enhances the reliability of the metasurface’s performance predictions.

**Table 2:** Equivalent circuit values

L_P_	C_P_	L_S_	C_S_	w_∘_
0.072 nH	0.608 pF	0.213 nH	0.206 pF	26.6 GHz
**g**_*p*_	**g**_*s*_	**R**_∘_	**Δ**	
4.2041	0.5898	50 *Ω*	0.85	

## Theory and discussion

### Polarization conversion ratio

To assess the cross PCR, one can express the PCR for the *y*-polarized and *x*-polarized incident EM waves using these coefficients as follows:

2a $${{\mbox{PCR}}}_{xy}=\frac{{{\mbox{R}}}_{xy}^{2}}{{{\mbox{R}}}_{xy}^{2}+{{\mbox{R}}\,}_{yy}^{\!\!2}},$$

2b $${{\mbox{PCR}}}_{yx}=\frac{{{\mbox{R}}}_{yx}^{2}}{{{\mbox{R}}}_{yx}^{2}+{{\mbox{R}}\,}_{xx}^{\!\!2}}.$$

Equations (2a) and (2b) express the cross PCR for waves with electric field components in the *y* and *x* directions, respectively. According to these equations, if R_*yx*_ and R_*xy*_ approach 1, and R_*xx*_ and R_*yy*_ approach 0, the PCR will be 1.

On the other hand, for RHCP and LHCP incident EM waves, the LHCP and RHCP conversion capabilities are determined as

3a $${{\mbox{PCR}}}_{-+}=\frac{{{\mbox{R}}}_{-+}^{2}}{{{\mbox{R}}}_{-+}^{2}+{{\mbox{R}}\,}_{++}^{\!\!2}},$$

3b $${{\mbox{PCR}}}_{+-}=\frac{{{\mbox{R}}}_{+-}^{2}}{{{\mbox{R}}}_{+-}^{2}+{{\mbox{R}}\,}_{--}^{\!\!2}}.$$

In these expressions, the  − and + indices denote left-handed and right-handed polarization, respectively. Equations (3a) and (3b) articulate the cross PCR for the RHCP and LHCP incident EM waves, respectively. Furthermore, in accordance with Equations (3a) and (3b), if R_+−_ and R_−+_ tend toward 1, and R_−−_ and R_++_ tend toward 0, the PCR_−+_ and PCR_+−_ will be 1.

### Circular polarization conversion

To assess the effectiveness of CP conversion for the *y*-polarized incident EM wave, the AR acts as a key indicator of CP, signifying its presence when it stays at or below 3 dB, denoted as *AR* ≤ 3 dB, as highlighted in^27^. The AR is defined as:

4 $$\,{{\mbox{AR}}}\,=\frac{1}{2}{\left(\frac{| {{\mbox{R}}}_{yy}{| }^{2}+| {{\mbox{R}}}_{xy}{| }^{2}+\sqrt{a}}{| {{\mbox{R}}}_{yy}{| }^{2}+| {{\mbox{R}}}_{xy}{| }^{2}+\sqrt{a}}\right)}^{\frac{1}{2}},$$

where *a* = ∣R_*yy*_∣^4^+ ∣R_*xy*_∣^4^ + 2∣R_*yy*_∣^2^∣R_*xy*_∣^2^cos(2Δ*ϕ*).

The ellipticity value provides valuable insights into LHCP, RHCP and is determined using Stokes parameters, as expressed in Equations (5). These equations break down into components:

5a $${{\mbox{S}}}_{0}=| {{\mbox{R}}}_{yy}{| }^{2}+| {{\mbox{R}}}_{xy}{| }^{2},$$

5b $${{\mbox{S}}}_{1}=| {{\mbox{R}}}_{yy}{| }^{2}-| {{\mbox{R}}}_{xy}{| }^{2},$$

5c $${{\mbox{S}}}_{2}=2| {{\mbox{R}}}_{yy}| | {{\mbox{R}}}_{xy}| \cos (\Delta \phi ),$$

5d $${{\mbox{S}}}_{3}=2| {{\mbox{R}}}_{yy}| | {{\mbox{R}}}_{xy}| \sin (\Delta \phi ),$$

By utilizing the Stokes Parameters, the CP ratio is determined through the ellipticity value, denoted as $e=\frac{{{\mbox{S}}}_{3}}{{{\mbox{S}}}_{0}}$. For CP, the R_*yy*_ and R_*xy*_ values for the incoming wave in the *y*-direction must be approximately equal, and the phase difference Δ*ϕ* between R_*yy*_ and R_*xy*_ must be nearly  ∓ 90° degrees. Notably, the ellipticity value takes ∓1 values for these conditions. Therefore, LHCP occurs when *e* = − 1 and Δ*ϕ* = −90°, while RHCP occurs when *e* = +1 and Δ*ϕ* = +90°.

## Simulation, and discussion

### Linear polarization

The metasurface polarization converter was designed utilizing CST Microwave Studio^*Ⓡ*^, with the frequency domain Floquet mode chosen for unit cell boundary conditions within the software. Due to the anisotropic characteristics of the reflective polarization converter, incident *y*-polarized waves upon such a structure undergo reflection involving both co-reflection (R_*yy*_) and cross-reflection R_*xy*_ components.

**Fig. 4:**
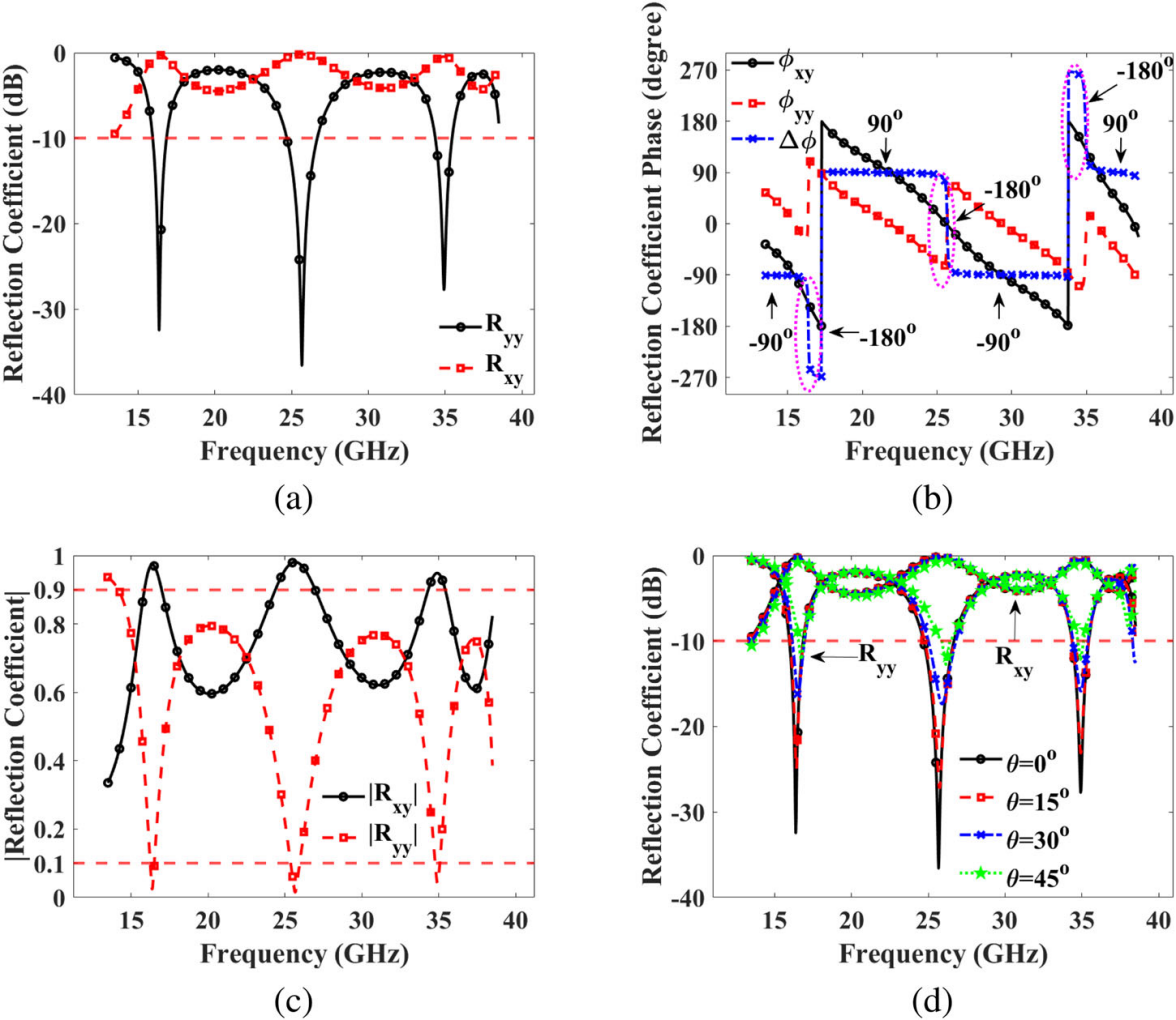
Simulation results. **a** co-reflection (R_*yy*_) and cross-reflection (R_*xy*_) coefficients in dB (**b**) the phase of co- and cross-reflection coefficients in degree (**c**) the magnitude of co- and cross-reflection coefficients (**d**) co- and cross-reflection coefficients (dB) under normal, and oblique incident electromagnetic wave.

In Fig. 4a, the co-reflection and cross-reflection coefficients are presented in dB. The co-reflection coefficients are below  −10 dB, while the cross-reflection coefficients are above  − 1 dB within the frequency ranges of 15.97-16.85 GHz, 24.70-26.65 GHz, and 34.37-35.45 GHz. Moreover, relative phase difference between co-reflection R_*yy*_ and cross-reflection R_*xy*_ is also observed. The relative phase difference is mathematically defined as

6 $$\Delta \phi =\arg ({{\mbox{R}}}_{xy})-\arg ({{\mbox{R}}}_{yy}),$$

The significance of polarization becomes evident through Eq. (6), showing that when Δ*ϕ* = 0, ±*π*, it is characterized as LP^18^. Analyzing Fig. 4b, it becomes apparent that at 16.37 GHz, 25.75 GHz, and 34.92 GHz, the phase differences Δ*ϕ* is approximately -180° in these three bands, i.e., 15.97–16.85 GHz, 24.70–26.65 GHz, and 34.37–35.45 GHz. A summary of the results for LP is given in Table 3. Moreover, if the amplitudes of R_*yy*_ and R_*xy*_ are equal, and $\Delta \phi =\pm \frac{\pi }{2}$, CP is achieved^15^. Figure 4a illustrates that the magnitudes of R_*yy*_ and R_*xy*_ are nearly equal between 14.57–15.65 GHz, 17.27–23.92 GHz, 27.47–33.85 GHz, and 35.87–38.32 GHz, while Fig. 4b shows that the phase difference Δ*ϕ* between these frequency ranges is -90°, 90°, -90°, and 90°, respectively. Therefore, it is evident that CP also occurs in these frequency regions.

**Table 3:** Linear polarization

**Freq (GHz)**	15.97–16.85	24.70–26.65	34.37–35.45
**Band**	Ku	K	Ka
**Resonance (GHz)**	16.37	25.75	34.92
**BW** (GHz)	0.88	1.95	1.08
**PCR**	≥95%	≥95%	≥95%
**AS**	≤45°	≤45°	≤45°
**Δ***ϕ*	−180°	− 180°	−180°

The amplitudes of the co-reflection and cross-reflection coefficients under normal incidence are depicted in Fig. 4c. Notably, as the amplitudes of the co-reflection coefficient approach 0.1, the amplitudes of the cross-reflection coefficient increase to 0.9 within the frequency ranges of 15.97–16.85 GHz, 24.70–26.65 GHz, and 34.37–35.45 GHz. Additionally, at the resonance regions of 16.37 GHz, 25.75 GHz, and 34.92 GHz, the co-reflection coefficient is almost zero, and the cross-reflection coefficient is nearly 1. Furthermore, Fig. 4d presents the co-reflection and cross-reflection coefficients in dB under oblique incidence up to 45°. As observed in Fig. 4d, the co-reflection and cross-reflection coefficients remain within the -10 dB and -1 dB regions up to 45°, respectively.

**Fig. 5:**
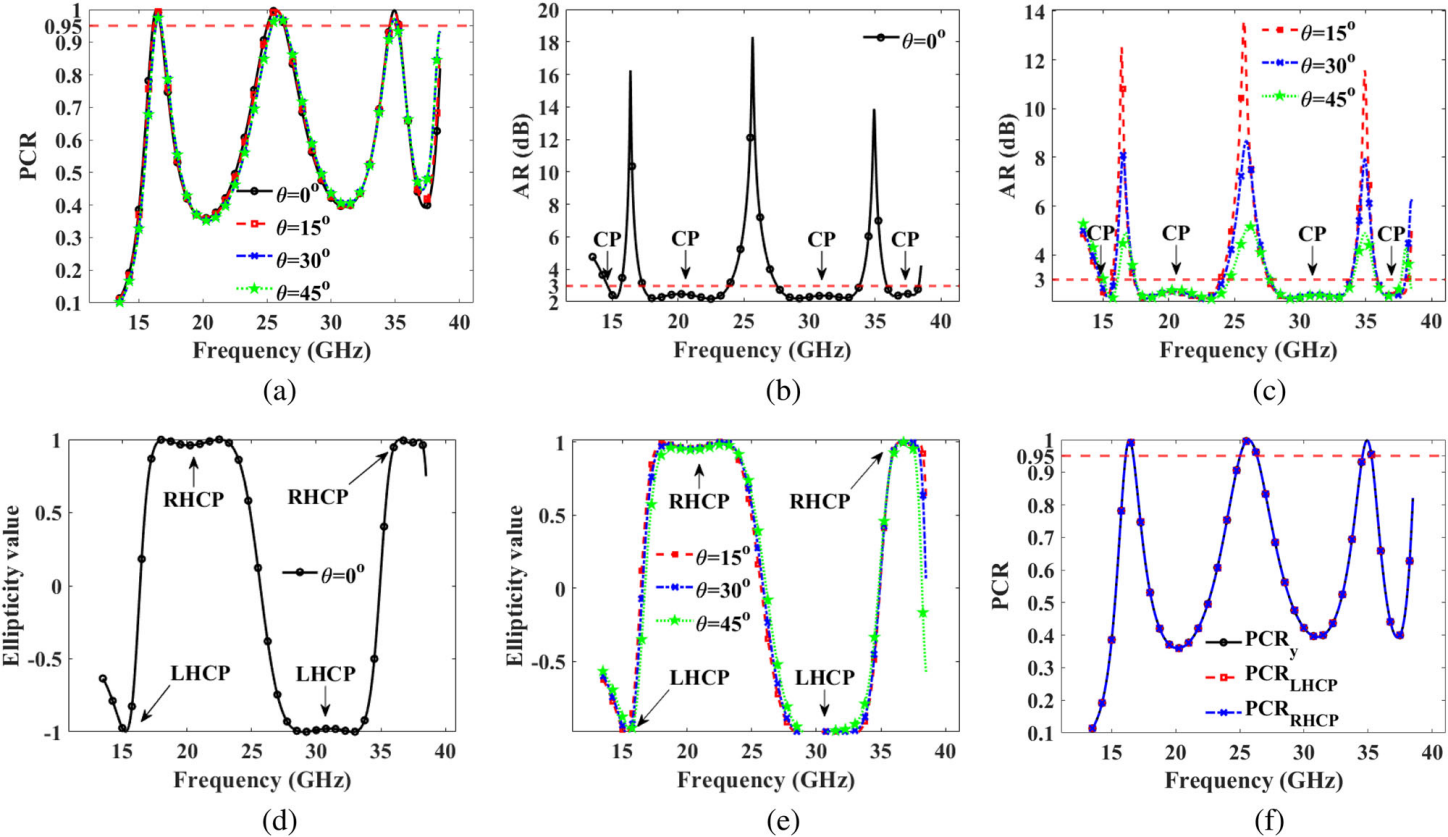
Simulation results. **a** polarization conversion ratio (PCR) under normal, and oblique incident electromagnetic (EM) waves (**b**) axial ratio (AR) under normal incident EM wave (**c**) AR under oblique incident EM waves (**d**) ellipticity under normal incident EM wave (**e**) ellipticity under oblique incident EM waves (**f**) PCR of *y*-polarized, left hand circular polarized, and right hand circular polarized incident EM waves.

The PCR of the proposed metasurface, as defined in Eq. (2), is illustrated in Fig. 5a under both normal and oblique incidence. As shown in Fig. 5a, the PCR value of the metasurface surpasses 95% under normal incidence in the frequency range from 15.97–16.85 GHz, 24.70–26.65 GHz, and 34.37–35.45 GHz. Additionally, under oblique angle conditions, over 95% PCR is achieved up to 45° degrees in the same frequency range.

### Circular polarization

The axial ratio (AR ≤ 3 dB) tells the presence of CP conversion in a particular frequency range. Figure 5b, c present the AR of the proposed polarization converter under normal and oblique incident EM waves, respectively. The AR remains below 3 dB from 14.57–15.65 GHz, 17.27–23.92 GHz, 27.47–33.85 GHz, and 35.87–38.32 GHz, indicating successful CP conversion. Furthermore, the AR remains below 3 dB under oblique angle conditions up to 45° in the aforementioned four frequency ranges. However, AR does not tells that which frequency range is LHCP or RHCP. This problem is solved by ellipticity whose value close to -1 tells the presence of LHCP, and +1 tells presence of RHCP. Figure 5d, e present the ellipticity of the proposed polarization converter under normal and oblique incident EM waves, respectively. The ellipticity value -1 tells the presence of LHCP in 14.57–15.65 GHz, and 27.47–33.85 GHz. Similarly, The ellipticity value +1 tells the presence of RHCP in 17.27–23.92 GHz, and 35.87–38.32 GHz. The comparison of Fig. 5b and d shows that the frequency ranges having AR ≤ 3 dB, the same frequency ranges are having ellipticity -1 or +1. Thus, ellipticity is well supporting the AR criteria.

Figure 5c depicts the ellipticity value across the same frequency bandwidth. It is evident that the ellipticity is -1 from 14.57–15.65 GHz and 27.47–33.85 GHz, and Fig. 4b shows that the phase difference Δ*ϕ* is -90° in both frequency ranges. Consequently, LHCP is achieved in these bandwidths. Additionally, Fig. 5c illustrates that the ellipticity is +1 from 17.27–23.92 GHz and 35.87–38.32 GHz, and Fig. 4b shows that the phase difference Δ*ϕ* is +90° in both frequency ranges. Hence, RHCP is realized at these bandwidths. Moreover, the ellipticity value remains close to  ∓1 under oblique angle conditions up to 45° in the aforementioned four frequency ranges. Figure 5d presents the PCR of the proposed metasurface for *y*-polarized, LHCP, and RHCP incident EM waves. As observed in Fig. 5d, RHCP and LHCP are also provided by the LHCP and RHCP incident EM waves for the four frequency ranges. A summary of the results for CP is provided in Table 4.

**Table 4:** Circular polarization

**Freq (GHz)**	14.57-15.65	17.27-23.92	27.47-33.85	35.87-38.32
**Band**	Ku	Ku, K	Ka	Ka
**BW (GHz)**	1.08	6.65	6.38	2.45
**AR** (dB)	≤3	≤3	≤3	≤3
**Ellipticity**	− 1	+ 1	− 1	+ 1
**Δ***ϕ*	− 90°	+ 90°	− 90°	+ 90°
**AS**	≤45°	≤45°	≤45°	≤45°
**Type of CP**	LHCP	RHCP	LHCP	RHCP

### Design evolution

The design process encompasses three key steps: (1) crafting the outer-ring with a singular slot; (2) creating the inner-ring with a rectangular slot and orienting it at -45°; and (3) fine-tuning the parameters. The primary aim of the initial step is to ensure the rotation of the *y*-polarized incident EM wave into its orthogonal *x*-polarized reflected EM wave, achieving LP and CP in lower and middle frequency ranges. The role of the inner-ring is to disrupt polarization states, thereby achieving LP and CP in the higher frequency range. Ultimately, the structure undergoes optimization to realize LP reflection EM waves in three bands and CP reflection EM waves in four distinct sub-bands.

**Fig. 6:**
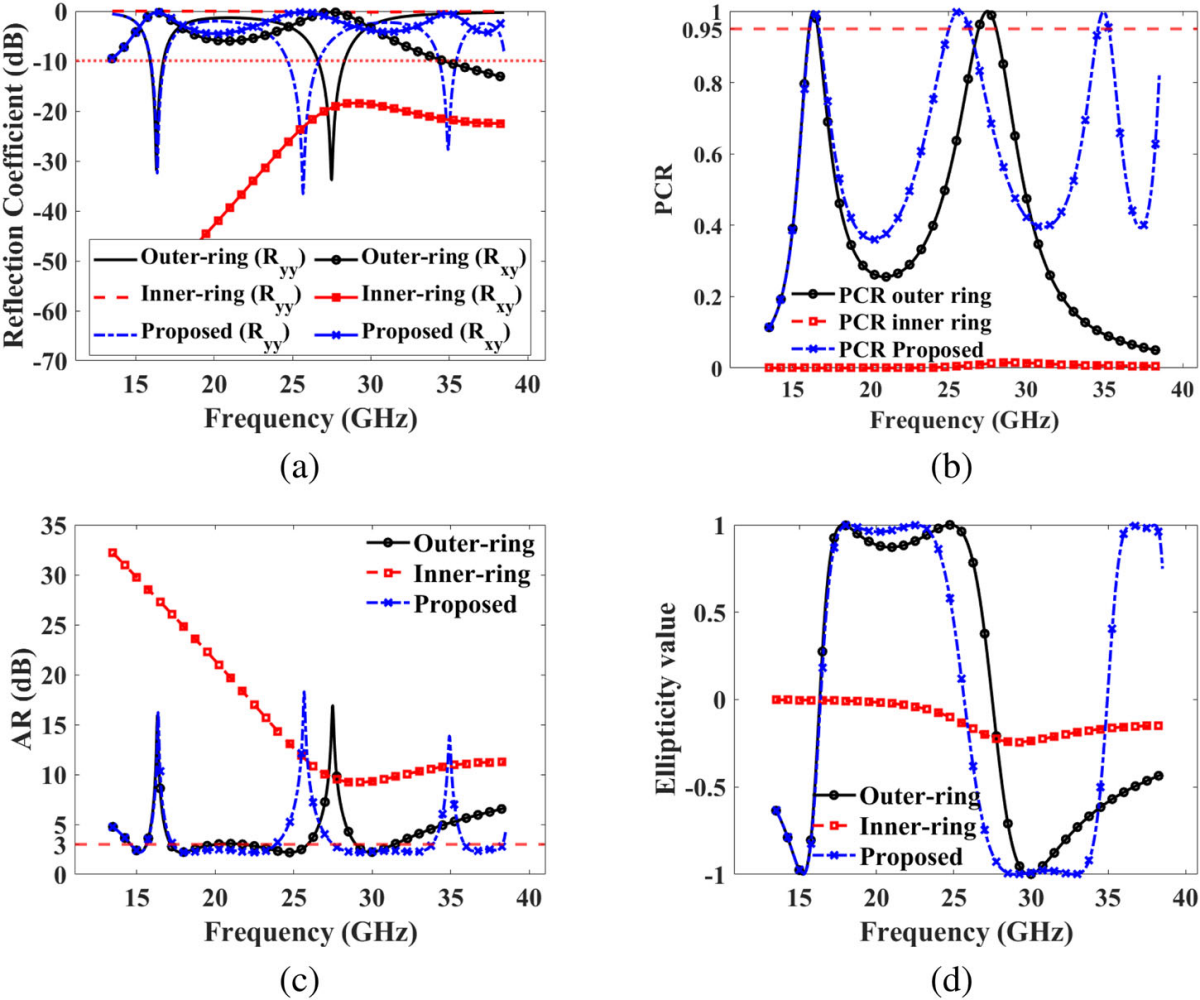
Design evolution. **a** co-reflection (R_*yy*_) and cross-reflection (R_*xy*_) coefficients of outer-ring, inner-ring, and proposed unit cell (**b**) polarization conversion ratio of outer-ring, inner-ring, and proposed unit cell (**c**) axial ratio of outer-ring, inner-ring, and proposed unit cell (**d**) ellipticity of outer-ring, inner-ring, and proposed unit cell.

The reflection coefficients, encompassing both co-polarization and cross-polarization, along with the PCR of the outer-ring, inner-ring, and the proposed structure, are portrayed in Fig. 6a, b. The outer ring displays co-polarization (R_*yy*_) below -10 dB within the frequency bands of 15.95–16.75 GHz and 26.65–28.30 GHz, attaining a PCR ≥ 0.90 in this range. This performance peaks at 100% at the resonance frequencies of 16.32 GHz and 27.5 GHz. Conversely, the inner-ring exhibits co-polarization (R_*yy*_) below -0.10 dB from 24.57–33.75 GHz. Simultaneously, the inner-ring demonstrates cross-polarization (R_*xy*_) above -20 dB within the 24.57–33.75 GHz range. Thus, the co- and cross-polarized reflection coefficients of the inner-ring are designed to perturb polarization states and achieve LP and CP in the higher frequency range. Thus, outer ring contribute to wideband LP conversion for *y*-polarized incident EM wave. When the outer-ring and inner-ring are amalgamated into a single metasurface, the proposed structure exhibits co-polarization (R_*yy*_) below -10 dB in three frequency ranges from 15.97–16.85 GHz, 24.70–26.65 GHz, and 34.37–35.45 GHz. It achieves a PCR ≥ 0.90 in this range, peaking at 100% at the resonance frequencies of 16.37 GHz, 25.75 GHz, and 34.92 GHz.

Figure 6c illustrates the variation of AR with respect to frequency for the outer-ring, inner-ring, and the proposed structure. Specifically, Fig. 6c reveals that the AR remains below 3 dB within three frequency ranges: 14.62–15.62 GHz, 17.17–25.9 GHz, and 29.00–31.40 GHz, when considering the outer-ring alone. Notably, the AR surpasses 9 dB across the broader range of 13.5–38.5 GHz for the inner-ring. Thus, outer ring contribute to wideband CP conversion for *y*-polarized incident EM wave. However, upon the amalgamation of the outer-ring and inner-ring into a unified metasurface, the proposed structure achieves an AR below 3 dB in four distinct frequency ranges: 14.57–15.65 GHz, 17.27–23.92 GHz, 27.47–33.85 GHz, and 35.87-38.32 GHz. This signifies the successful achievement of CP across the aforementioned four frequency ranges.

Figure 6d presents the variation of ellipticity (*ϵ*) values across the frequency spectrum for the outer-ring, inner-ring, and the proposed structure. In the case of the outer-ring, LHCP is evident within the frequency bands of 14.62–15.62 GHz and 29.00–31.40 GHz, and RHCP is observed from 17.17–25.9 GHz. This distinction arises from the *ϵ* values being  −1 and  +1 in the specified frequency bands, aligning with LHCP and RHCP, respectively. In contrast, the inner-ring does not exhibit distinct LHCP or RHCP characteristics. However, upon the amalgamation of the outer-ring and inner-ring into the unified metasurface, the proposed structure displays LHCP from 14.57–15.65 GHz and 27.47–33.85 GHz, as well as RHCP from 17.27–23.92 GHz and 35.87–38.32 GHz. This arises from the *ϵ* values closely approximating  −1 and  1 in the respective frequency bands, establishing LHCP and RHCP properties.

The unit cell design, illustrated in Fig. 1a, is versatile and can be tailored to meet the frequency requirements of various satellite communication bands. The design parameters for shifting the center frequency and bandwidth for LP and CP, including LHCP and RHCP, are as follows:

- The design of the outer and inner rings is crucial for achieving LP and CP, including LHCP and RHCP.
- The length L and substrate thickness h of the unit cell are critical in shifting the LP and CP bands to lower or higher frequencies. The relationship between length and center frequencies is inversely proportional, as is the case for substrate thickness.
- Adjusting R_1_ and R_2_ can marginally shift the LP and CP bands to lower or higher frequencies. The relationship between R_1_ and center frequency for the LP and CP bands is also inversely proportional, as is the case for R_2_
- Modifying the lengths *x*, *y*, and slot *s* helps achieve stable PCR, LHCP, and RHCP for oblique *y*-polarized incident EM waves.

## Conclusion

This research introduces an ultra-thin reflective metasurface tailored for satellite communication, and remote sensing applications in the Ku, K, and Ka bands. Operating within distinct sub-bands, the metasurface demonstrates cross-polarization effectiveness in the Ku band (15.97–16.85 GHz), K band (24.70–26.65 GHz), and Ka band (34.37–35.45 GHz). Additionally, it successfully achieves left-handed circular polarization (LHCP) in two sub-bands and right-handed circular polarization (RHCP) in two sub-bands. The proposed design attains a 95% polarization conversion ratio (PCR) under both normal and oblique incidences up to 45°. Characterized by a slim profile with a thickness of 0.64 mm, the design caters to applications requiring reduced thickness. Utilizing CST software for simulation and traditional PCB printing techniques for fabrication, the real-time measurements align closely with simulation outcomes.

## Data Availability

Correspondence and requests for data should be addressed to Qammer H. Abbasi, and Humayun Zubair Khan.
